# Possible Effects of Manual Stripping on Stress Response and Reproductive Status in Captive Female *Salmo carpio*

**DOI:** 10.3390/ani16213355

**Published:** 2026-10-24

**Authors:** Lorenzo Rossi, Federico Conti, Matteo Zarantoniello, Elia Ciani, Francesca Ciutti, Giulia Chemello, Giorgia Gioacchini, Ike Olivotto, Filippo Faccenda

**Affiliations:** 1 Aquaculture and Hydrobiology Unit, Centro Trasferimento Tecnologico-Fondazione Edmund Mach, 38098 Trento, Italy; lorenzo.rossi@fmach.it (L.R.); elia.ciani@fmach.it (E.C.); francesca.ciutti@fmach.it (F.C.); filippo.faccenda@fmach.it (F.F.); 2 Department of Life and Environmental Sciences, Università Politecnica delle Marche, 60131 Ancona, Italy; federico.conti@staff.univpm.it (F.C.); g.chemello@staff.univpm.it (G.C.); giorgia.gioacchini@staff.univpm.it (G.G.); 3 Department of Agricultural, Food and Environmental Sciences, Università Politecnica delle Marche, 60131 Ancona, Italy

**Keywords:** salmonids, histology, fish welfare, reproduction, aquaculture, stress response

## Abstract

Captive breeding programs are essential for conserving the endangered salmonid *Salmo carpio*. However, standard hatchery operations rely on manual stripping, whose effects on Carpione reproductive physiology and welfare remain poorly understood. This preliminary study investigated the physiological and reproductive responses of captive female *S. carpio* to manual stripping. Forty-five mature females were assigned to three groups: pre-stripping (PRE), 1 day post-stripping (POST1), and 21 days post-stripping (POST21). Plasma concentrations of 17β-estradiol, testosterone, progesterone, cortisol, glucose, and antioxidant enzyme activities (SOD, CAT, GPx) were assessed alongside ovarian histology. While no effects were detected on circulating steroid or glucose levels, POST21 females showed significantly higher cortisol and increased SOD and CAT activities than PRE and POST1 fish (*p* < 0.05), while both POST1 and POST21 showed higher GPx activity compared to PRE, indicating a delayed modulation of stress response and oxidative challenge after the stripping event. Ovarian histology revealed no evident histopathological alterations. PRE group ovaries were characterized by the presence of primary growth and tertiary vitellogenic oocytes. Primary growth oocytes were identified in all the experimental groups. Secondary vitellogenic oocytes, together with post-ovulatory follicles and atretic oocytes, were observed at POST1. At POST21, histological examination revealed primary growth and early vitellogenic oocytes, while mature oocytes were also identified macroscopically, supporting the persistence of ovarian reproductive activity during the reproductive season. Overall, females maintained ovarian activity throughout the examined period, while delayed systemic stress during post-spawning recovery was observed in the POST21 group. Broodstock management should therefore include adequate recovery periods and reduce handling to support welfare and reproductive performance.

## Simple Summary

The Carpione del Garda (*Salmo carpio*) is an endangered fish endemic to Lake Garda whose survival depends heavily on captive breeding programs. In hatcheries, eggs are collected manually by stripping, a procedure that may induce stress and affect fish welfare. This study assessed the response of female *S. carpio* to stripping by measuring reproductive hormones, stress indicators (cortisol and glucose), and antioxidant defenses before stripping, 1 day after, and 21 days after. The results indicated that no differences were detected in ovarian integrity or reproductive hormone levels, while a delayed increase in cortisol and antioxidant enzyme activity was observed 21 days after the stripping procedure. These findings suggest that the post-spawning recovery phase imposes prolonged physiological demands, highlighting the need for management practices that reduce handling and improve fish welfare.

## 1. Introduction

Salmonid aquaculture represents a major component of European fish production, accounting for more than 17% of EU aquaculture value in 2023, with salmon and trout as the main cultured species [1]. Despite its economic importance, rainbow trout (*Oncorhynchus mykiss*, Walbaum) production has shown limited expansion over the last decade, increasing by only 8% between 2011 and 2020 [2]. In 2023, Italy was the fourth country in terms of aquaculture production (12%) at the EU level, with rainbow trout farming accounting for 27% of the total national production, with an ex-farm value of 207 million euros [3]. Previously published data showed a production decline of approximately 5%, despite an increasing market value (+13%), highlighting the need for diversification strategies to improve sector resilience and sustainability [2]. In this context, alternative salmonid species may represent a viable opportunity to enhance production without major changes to existing farming systems.

In Northern Italy, salmonid aquaculture is traditionally based on rainbow trout and Arctic char (*Salvelinus alpinus* L.). Increasing attention has recently been given, especially in the regions bordering the lake, to the Carpione del Garda (*Salmo carpio* L.), an endemic and endangered salmonid restricted to Lake Garda [4,5]. The species is highly specialized, inhabiting deep pelagic waters below 150 m. Reproduction involves seasonal migrations from the central-southern basin toward northern spawning grounds at 150–200 m depth on gravelly sandy substrates (200–500 m offshore) [6]. Sexual maturity in wild conditions occurs at approximately 3 years in males and 4 years in females [7]. Spawning phenology remains debated, although a main winter peak (December-February) and a secondary summer event (July-August) are generally recognized [5] with some females spawning twice within a year, likely due to rapid post-spawning vitellogenesis [7]. After spawning, adults migrate to the southern basin for feeding [6].

Despite its conservation relevance, *S. carpio* is classified as endangered by the IUCN [4] and remains poorly characterized under captive conditions. Although experimental rearing has been performed for decades, species-specific reproductive and husbandry protocols are still lacking. Current practices are mainly extrapolated from other salmonids, particularly brown trout and rainbow trout, and do not account for the species’ specific physiological traits. Nutritional knowledge is still limited, although preliminary grow-out dietary responses have been reported [8]. Overall, major gaps persist, especially in reproductive physiology under farming conditions.

A major constraint in salmonid aquaculture is the lack of spontaneous spawning in captivity, requiring the collection of gametes by manual stripping of broodstock [9]. In females, stripping consists of abdominal pressure to induce egg release and represents a critical procedure with direct implications for animal welfare and post-spawning survival. In addition to mechanical stress, which can damage the skin protective barrier, stripping commonly involves air exposure, inducing oxidative stress and increasing mortality risk [9,10]. In fact, air exposure is a well-established acute stressor in fish, driving strong systemic responses and increasing allostatic load [11]. These effects are particularly relevant in females, where reproductive success and survival depend strongly on physiological status [12]. Therefore, stripping represents a major welfare bottleneck and a key constraint for reproductive efficiency in salmonid broodstock management [9,13]. Previous studies in salmonids have investigated the effects of different stripping procedures on broodstock and reproductive outcomes, including egg output, post-spawning female survival, and egg-quality-related parameters [10,14]. However, information on ovarian status following egg collection and its relationship with the physiological condition of broodstock remains limited.

Like for other vertebrates, reproduction in salmonids is regulated by the hypothalamus-pituitary-gonadal (HPG) axis [15]. Gonadotropin-releasing hormone (GnRH) stimulates follicle-stimulating hormone (FSH) and luteinizing hormone (LH) secretion, controlling steroidogenesis and gonadal maturation [16]. In females, the principal reproductive steroids include testosterone and 17β-estradiol (E2), which mediate the effects of FSH during oocyte growth and vitellogenesis, whereas 17α,20β-dihydroxyprogesterone (DHP) acts as the maturation-inducing steroid, mediating the effects of LH during final oocyte maturation [13,17,18,19]. Oocyte development requires high energetic investment, particularly during vitellogenesis, when resources are allocated to yolk synthesis [12,17]. Consequently, reproductive output is tightly dependent on the fish’s physiological condition and is highly sensitive to environmental and stress-related disturbances [20,21].

Stress responses in fish are mediated by the hypothalamus-pituitary-interrenal (HPI) axis, resulting in cortisol and catecholamine release [22]. In particular, cortisol is the main systemic stress indicator and modulates metabolism, immunity, growth, behavior, and reproduction through crosstalk with the HPG axis [22]. Moreover, stress-induced metabolic adjustments may include increased glucose mobilization to satisfy the energetic requirements of reproductive processes, as evidenced by elevated plasma glucose levels reported in sockeye salmon [23]. However, glucose itself is not a reliable stress biomarker in teleosts due to inconsistent and erratic results reported in the literature [24].

Given the ecological specialization of *S. carpio*, its adaptation to deep-water habitats, and its unusual reproductive strategy, differences in endocrine regulation and stress responsiveness compared with model salmonid species are to be expected. However, knowledge of its reproductive endocrinology and physiological responses to manual stripping remains extremely limited. This lack of information represents a major obstacle to the development of effective broodstock management practices and hinders efforts to optimize animal welfare and reproductive performance in conservation-oriented aquaculture programs.

The present study therefore investigated captive-reared female *S. carpio* reproduction, focusing on the relationship between reproductive status and physiological responses during the period following manual stripping. Reproductive hormones and stress indicators were assessed together with egg production and ovarian histology. Histological analysis was included to characterize ovarian reproductive status during the post-stripping period and to assess the presence of evident histopathological alterations. The ultimate goal was to provide a welfare-oriented physiological framework to support the development of optimized broodstock management strategies for this endangered salmonid.

## 2. Materials and Methods

### 2.1. Ethics

All experimental procedures involving animals were carried out in strict accordance with EU legal frameworks relating to the protection of animals used for scientific purposes (Directive 2010/63/EU) and were approved by the Ethics Committee of Fondazione Edmund Mach (FEM) (protocol n° 0001566, 26 February 2021).

### 2.2. Broodstock Maintenance, Sampling Period, and Procedures

All experimental procedures were conducted at the Aquaculture Facility of Fondazione Edmund Mach (FEM), San Michele all’Adige, Italy (46°11′32.9″ N, 11°08′05.1″ E). A total of 750 sexually mature *Salmo carpio* broodstock (approximately 3 years old; mean body weight 400.5 ± 57.3 g) were maintained under natural photoperiod and ambient freshwater temperature conditions (12–14 °C) in three 16 m^3^ rearing tanks (250 fish per tank). Dissolved oxygen levels were continuously maintained near saturation. Broodstock fish were fed a commercial salmonid broodstock diet (REPRO FM 6 Floating, Veronesi, Verona, Italy) according to standard husbandry practices, with feed supplied daily to apparent satiation. All fish were individually identified using subcutaneous passive integrated transponder (PIT) tags (Biomark, Boise, ID, USA), allowing individual tracking and sex identification throughout the study. The reproductive season extended for approximately 160 days, from mid-February to the end of July.

During the peak spawning period (April–May), 20 mature females were randomly collected from each broodstock tank and randomly redistributed among three 700 L experimental tanks (20 fish per tank). Fish were maintained under the same environmental conditions and feeding regime as the broodstock population. After a three-week acclimation period (May), 15 females were randomly selected from each experimental tank (45 fish in total) and assigned to three experimental groups (*n* = 15 per group; 5 fish from each tank): PRE, POST1, and POST21.

Fish assigned to the PRE group (*n* = 15, 5 fish from each experimental tank) were anaesthetized with tricaine methanesulfonate (MS-222; 80 mg L^−1^) and euthanized by overdose (≥200 mg L^−1^) before sampling, without undergoing stripping.

All the remaining fish were anaesthetized using the same protocol and subjected to manual stripping by gentle abdominal pressure. The stripping procedure, including air exposure, lasted approximately 60 s per fish. Following stripping, fish were assigned to either POST1 or POST21 (*n* = 15, 5 fish from each experimental tank) and maintained under the same environmental conditions and feeding regime as the broodstock. Fish in the POST1 and POST21 groups were euthanized and sampled 1 and 21 days after stripping, respectively, using an overdose of MS-222 following the same anesthetic protocol adopted for the PRE group. At the end of the experiment, the remaining 15 females were returned to the broodstock tanks and were not included in the experiment.

No additional unstripped control group was included at 21 days since, under captive conditions, sexually mature *S. carpio* females do not necessarily undergo spontaneous oviposition. Maintaining mature females without stripping would have introduced additional stressors, such as prolonged egg retention and related endocrine and metabolic changes, representing a confounding factor. Since manual stripping is a standard broodstock management practice, the PRE group was considered the appropriate physiological baseline to evaluate post-stripping responses and recovery over time, minimizing the introduction of additional factors unrelated to the handling protocol.

Throughout the recovery period, tanks were inspected daily for mortality, and fish were fed according to the broodstock feeding protocol described above.

From all the fish of each experimental group, blood samples were collected from the caudal vein using sterile syringes containing EDTA as an anticoagulant. A new syringe was used for each specimen to avoid cross-contamination. Blood samples were centrifuged on-site at 1500× *g* for 15 min (Eppendorf^®^ Centrifuge MiniSpin G, Sigma-Aldrich, St. Louis, MO, USA), and plasma was aliquoted (500 µL) and stored at −80 °C until hormonal and biochemical analyses (no hemolysis occurred).

Following blood collection, ovaries were dissected, immediately fixed for histological analysis in formaldehyde/glutaraldehyde solution [NaH_2_PO_4_-H_2_O + NaOH + Formaldehyde (36.5%) + Glutaraldehyde (25%) + H_2_O] with a final concentration of 4.03% (*v*/*v*) and 1% (*v*/*v*) of formaldehyde and glutaraldehyde, respectively. All samples were fixed for 48 h at 4 °C under identical conditions across experimental groups, then rinsed and stored in 70% ethanol until histological processing.

Eggs obtained after stripping of POST1 and POST 21 fish were collected and counted, and egg volume was measured using a graduated cylinder. Egg density (eggs L^−1^) was calculated as the number of eggs counted divided by the measured egg volume (L).

### 2.3. Hormone Extraction and Quantification

Circulating 17β-estradiol, testosterone, progesterone, and cortisol were quantified in the plasma of each fish (*n* = 15 for each experimental group), using commercially available Enzyme-linked Immunosorbent Assay (ELISA) kits (Cayman Chemical, Ann Arbor, MI, USA: Estradiol N.501890; Testosterone N.582701; Progesterone N.582601; Cortisol N.500360), according to the manufacturer’s instructions with minor modifications (e.g., adjustments in the dilution factor to ensure measurements fell within the linear range of standard curve). Steroid hormones were extracted from plasma using organic solvent extraction procedures optimized for each analyte.

Briefly, for 17β-estradiol extraction, plasma samples were mixed with methanol (1:4, *v*/*v*), incubated for 10 min, and centrifuged at 2000× *g* for 10 min; the supernatant was collected and evaporated under a nitrogen stream, and residues were reconstituted in 300 µL ELISA buffer. Testosterone and progesterone were both extracted using diethyl ether (1:5 and 1:4, *v*/*v*, respectively), repeated three times; pooled organic phases were evaporated to dryness and reconstituted in 500 µL ELISA buffer. Cortisol was extracted using ethyl acetate (1:4, *v*/*v*), repeated three times, followed by evaporation and reconstitution in 500 µL ELISA buffer.

All samples were analyzed in duplicate on 96-well microplates following kit protocols. Optical density was measured using a Synergy HTX microplate reader (BioTek, Winooski, VT, USA) at 405–420 nm over a 120 min period. Hormone concentrations were subsequently determined from the resulting standard curves using a logit-log transformation, as recommended by the manufacturer. Assay validation was performed according to Metcalfe et al. [25]. Briefly, parallelism was assessed by comparing the dose–response curves of the samples at different dilutions with the corresponding standard curves obtained from the Cayman Chemical ELISA kits (R^2^ close to 1 for all the tests; B/B_0_ values between 20 and 80% were considered). Accuracy was assessed by spike and recovery (mean recoveries 80–120%) used as correction factors. Precision was assessed with an intra-assay coefficient of variation (CV) < 10% and an inter-assay CV < 15%.

### 2.4. Plasma Glucose Assay

Plasma glucose levels were quantified in the plasma of each fish (*n* = 15 for each experimental group), using a commercial colorimetric assay kit (Item No. 10009582; Cayman Chemical, USA). The standard curve was generated using concentrations of 0, 2.5, 5, 7.5, 10, 15, 20, and 25 mg dL^−1^. Samples and standards were assayed in duplicate, with absorbance measured at 510 nm using a Synergy HTX (Biotek Instruments Inc., Winooski, VT, USA) microplate reader. Final glucose concentrations were determined via linear interpolation from the standard curve.

### 2.5. Antioxidant Enzyme Assays

Superoxide dismutase (SOD) activity (*n* = 15 plasma samples for each experimental group) was measured by the xanthine oxidase/cytochrome c method Crapo et al. [26]. This assay is based on the spectrophotometric measurement of cytochrome c reduction by superoxide anion (xanthine oxidase/hypoxanthine reaction), which was monitored at 550 nm. Measurements were performed using a 10 mm light path cuvette and a NanoPhotometer^®^ P330 (Implen, GmbH, Munich, Germany). The reaction mixture contained 46.5 mM potassium phosphate buffer (pH 7.8), 0.1 mM EDTA, 0.05 mM xanthine, 0.02 mM cytochrome c, and 2.5 µU of xanthine oxidase (XO). Enzymatic activity is expressed as U/mg of protein (one unit, U, of SOD corresponds to the amount of enzyme that produces 50% inhibition of cytochrome c reduction).

Catalase (CAT) activity (*n* = 15 plasma samples for each experimental group) was assayed following Aebi [27]. Aliquots (10 µL) of plasma were mixed with 30 mM H_2_O_2_ in 50 mM phosphate buffer (pH 7.8) and absorbance decrease was recorded every 10 s for 3 min at 240 nm in a quartz cuvette. Results were expressed as U/mg of protein (one U of CAT corresponds to the decomposition of 1 µmol H_2_O_2_ min^−1^).

Glutathione peroxidase (GPx) activity (*n* = 15 plasma samples for each experimental group) was measured in a 96-well plate following the method described in Sattar et al. [28]. Briefly, the reaction mixture contained 100 μL of reduced glutathione (500 μM) prepared in phosphate buffer (100 mM; pH = 7.0) and 10 μL of supernatant. The reaction was started by adding 40 μL of hydrogen peroxide (1.5 mM) and incubated for 10 min at 37 °C. Then, the reaction was stopped with 65 μL of 2% (*w*/*v*) sodium hydrogen phosphate (Na_2_HPO_4_) solution and 35 μL of Ellman’s reagent, and the absorbance was measured at 412 nm using a microplate reader (Synergy HTX, Biotek Instruments Inc., Winooski, VT, USA). GPx activity was expressed as U/mg of protein (one U of GPx corresponds to the oxidation of 1 µmol of reduced glutathione, GSH, to oxidized glutathione, GSSG, min^−1^, pH 7.0 and 37 °C) [29].

The protein content in plasma samples was determined according to Bradford [30] using bovine serum albumin as a standard.

### 2.6. Histological Analysis of Ovaries

Before histological processing, the ovaries were macroscopically examined to identify mature oocytes, which were readily visible to the naked eye and characterized by an orange-yellow coloration and a mean diameter of approximately 5 mm. Due to their large size, mature oocytes were not included in the ovarian portions selected for histological processing.

Ovarian portions (*n* = 15 for each experimental group) were repeatedly washed in 70% ethanol to remove any residual fixative solution and then stored in 70% ethanol for 24 h. Samples were subsequently dehydrated through a graded ethanol series (80, 95, and 100%), washed in xylene, and individually embedded in molten paraffin, which was then allowed to solidify in dedicated blocks, following the protocol described by Chemello et al. [31].

Paraffin-embedded samples were sectioned at 5 µm thickness using a microtome (Leica RM2125 RTS, Leica Microsystems GmbH, Nussloch, Germany). Sections were stained with Mayer’s hematoxylin and eosin Y (H&E) and then examined under an optical microscope (Zeiss Axio Imager.A2, Zeiss, Oberkochen, Germany). All ovarian samples were examined, and representative micrographs were selected for presentation.

All ovarian sections were evaluated by an experienced histological reader blinded to the experimental group allocation. Each slide was examined twice in two separate reading sessions. The experimental group allocation (PRE, POST1, and POST21) was disclosed only after all histological evaluations and classifications had been completed.

Ovarian histology was used for two complementary purposes. First, reproductive status was characterized based on established histological markers of ovarian development, including the occurrence of oocytes at different developmental stages, post-ovulatory follicles (POFs), and follicular atresia, according to Brown-Peterson et al. [32]. The assessment focused on identifying oocyte developmental stages and post-ovulatory structures rather than quantifying the relative abundance of individual oocyte stages or formally assigning reproductive phases to individual ovaries. Second, ovarian sections were screened for evident histopathological alterations according to established criteria for fish gonad histopathology. Severity grading was performed using a semi-quantitative approach to estimate the extent of each histomorphological alteration observed in the tissue sections, following the criteria described in the OECD Guidance Document on the Diagnosis of Endocrine-Related Histopathology in Fish Gonads [33]. The semi-quantitative severity grading system was applied individually to each ovarian sample (*n* = 15 per experimental group). The original OECD severity grading categories and their corresponding descriptions are reported in Table 1. The evaluation included preservation of normal ovarian tissue architecture and the occurrence of tissue necrosis, interstitial fibrosis, inflammatory cell infiltration, and evident degenerative alterations of oocyte morphology, including abnormal cytoplasmic vacuolization and alterations of the zona radiata [33,34]. Follicular atresia was recorded as a feature of ovarian reproductive status. Occasional atretic oocytes consistent with physiological ovarian remodelling were distinguished from pathological follicular degeneration and were not included in the histopathological severity grading [34].

### 2.7. Statistical Analysis

Data normality was assessed with the Shapiro–Wilk test and homogeneity of variances with Levene’s test. Group comparisons were performed by one-way ANOVA followed by Tukey’s post hoc multiple comparisons (Prism 8, GraphPad software version 8.0.2, San Diego, CA, USA). The biomarkers considered (17β-estradiol, testosterone, progesterone, cortisol, glucose, SOD, CAT, and GPx) were analyzed separately because they represent distinct physiological endpoints, and the analyses were defined a priori based on their respective biological roles. No formal correction for multiple comparisons was applied across biomarkers; therefore, the interpretation of the results could represent a possible limitation due to the low statistical power, and the analysis has also been kept focused on the biological relevance and consistency of the observed patterns rather than on isolated statistical significance. Egg production was compared between POST1 and POST21 groups, and an unpaired Student’s *t*-test was used. Tanks were considered as experimental units. The significance threshold was *p* < 0.05. Data are reported as mean ± standard deviation (SD).

## 3. Results

### 3.1. Egg Production Data

Females yielded an average of 0.14 ± 0.04 L and 0.13 ± 0.08 L of eggs (corresponding to approximately 8000 eggs L^−1^) for POST1 and POST21 groups, respectively, and no female fish mortality was observed during the whole experimental period. No significant differences were detected between the two groups in terms of eggs produced (*t* = 0.19; *df* = 4; *p* = 0.86).

### 3.2. Steroid Hormone Profiles

Plasma concentrations of 17β-estradiol, testosterone, and progesterone did not show significant differences among the experimental groups (Figure 1).

### 3.3. Plasma Cortisol

Figure 2 shows cortisol concentration measured in plasma of female Carpione at the three reproductive time points considered in this study. POST21 group showed a significantly higher cortisol level (*p* < 0.05) compared with both PRE and POST1, which did not show significant differences between them.

### 3.4. Plasma Glucose

Plasma glucose concentrations did not significantly differ among experimental groups (*p* > 0.05). Mean glucose levels (mg·dL^−1^) for PRE, POST1, and POST21 are shown in Figure 3.

### 3.5. Antioxidant Enzyme Activities

Figure 4 shows oxidative stress enzyme activities analyzed in plasma samples of female Carpione. SOD and CAT activities were significantly higher in POST21 group compared to both PRE and POST1 ones (*p* < 0.05), which did not show significant differences between them. Additionally, GPx activity was significantly higher in both POST1 and POST21 groups compared to the PRE group (*p* < 0.05).

### 3.6. Histology of Ovaries

Histological examination showed the presence of oocytes at different developmental stages throughout the experimental period (Figure 5). Macroscopic examination confirmed the presence of mature oocytes in ovarian tissue from all experimental groups, including POST21. As described in the Materials and Methods, these oocytes were not included in the histological sections. Samples from the PRE group exhibited the typical appearance of a mature ovary, containing primary growth (PG) and tertiary vitellogenic oocytes (Vtg3) (Figure 5A). POST1 ovaries contained PG and secondary vitellogenic oocytes (Vtg2), together with post-ovulatory follicles (POFs) and atretic oocytes (Figure 5B). POST21 ovaries also contained PG oocytes and primary vitellogenic oocytes (Vtg1) (Figure 5C). No evident histopathological lesions were observed in the ovarian sections examined in any experimental group. For all histomorphological alterations evaluated, each ovarian sample (*n* = 15 per experimental group) was individually classified as Not Remarkable according to the adopted semi-quantitative severity grading system. Normal ovarian tissue architecture was preserved, with no evidence of tissue necrosis, interstitial fibrosis, inflammatory cell infiltration, or evident degenerative alterations of oocyte morphology, including abnormal cytoplasmic vacuolization or alterations of the zona radiata.

## 4. Discussion

Carpione del Garda (*Salmo carpio*) is an endangered salmonid species endemic to Lake Garda (Italy), whose conservation currently depends on captive breeding programs. Results of the present study show that *S. carpio* exhibits a prolonged spawning season in captivity, with peak reproductive activity occurring in April–May, consistent with observations from wild populations, where spawning peaks have been reported before and after this period [7]. Furthermore, the present findings suggest precocious sexual maturation in *S. carpio*, with successful reproduction observed in 3-year-old females, compared with the ages at maturity previously reported in the literature (4 years). The earlier maturation observed in the present study may be attributed to the environmental conditions of captive rearing. In hatchery settings, continuous feed availability and the absence of long-distance trophic migrations are likely to reduce energetic expenditure, thereby supporting faster growth and earlier sexual maturation, as previously reported in other salmonids [35,36]. Moreover, egg production did not differ significantly between females sampled at POST1 and POST21 at the time of stripping.

Reproductive management in captivity of this species is largely based on protocols developed for other salmonids and mainly relies on manual gamete stripping and artificial fertilization [10]. However, these procedures do not consider the species-specific reproductive physiology of *S. carpio*, and little is known about their potential effects on reproductive function and animal welfare under farming conditions.

To address these gaps, Carpione mature females were sampled specifically before stripping (PRE) and after stripping (POST1 and POST21). The study aimed to characterize the physiological and reproductive status of females during the period following manual egg collection and to investigate possible welfare implications associated with current broodstock management practices.

In teleosts, including salmonids, circulating sex steroids play a central role in regulating gonadal maturation and spawning, and their concentrations typically fluctuate according to the reproductive cycle [37]. Estradiol and testosterone generally increase during vitellogenesis and ovarian maturation, reaching peak levels before ovulation. Additionally, final oocyte maturation is primarily regulated by the maturation-inducing steroid 17α,20β-dihydroxyprogesterone, whereas circulating progesterone can be used as a complementary endocrine indicator of reproductive status [38]. Following spawning, steroid hormone levels usually decline and reach their lowest seasonal values [39,40]. Therefore, circulating concentrations of estradiol, testosterone, and progesterone are commonly used as endocrine indicators of reproductive status and gonadal activity [17,18].

In the present study, no significant differences in circulating estradiol, testosterone, and progesterone concentrations were detected among PRE, POST1, and POST21 females. The maintenance of relatively stable steroid profiles throughout the sampling period suggests that females retained an active reproductive condition across all examined phases, as observed also in Atlantic salmon (*Salmo salar*) females during the reproductive season [38].

This pattern was consistent with the histological results, which revealed the presence of developing oocytes across all experimental groups, supporting the persistence of ovarian reproductive activity during the examined period. This is particularly relevant in *S. carpio*, in which stripping represents a discrete egg-collection event within a prolonged reproductive season rather than the end of ovarian activity. The occurrence of POFs and atretic follicles after stripping was also compatible with normal post-ovulatory ovarian remodelling. POFs are expected after ovulation, while follicular atresia is a physiological component of the teleost ovarian cycle and should not be interpreted *per se* as evidence of ovarian pathology [32,34].

The presence of primary growth and early vitellogenic oocytes in POST21 ovarian sections, together with the macroscopic identification of mature oocytes, supports the persistence of ovarian reproductive activity throughout the examined period, suggesting ongoing oocyte development during the reproductive season.

At the same time, the absence of evident histopathological lesions suggested that no major structural alterations of the ovary were detectable under the conditions examined.

Although stripping is routinely used in captive breeding programs, handling and manual gamete collection are recognized as potential stressors in fish and may interfere with reproductive physiology and animal welfare through activation of the HPI axis [41,42,43]. In teleosts, prolonged or intense stress can negatively affect steroidogenesis, gonadal function, spawning performance, and, in general, fish welfare status [43,44]. In this regard, a commonly adopted indicator of fish welfare and stress status is cortisol, since elevated cortisol concentrations can alter physiological functions, behavior, immune competence, and disease susceptibility [41]. Plasma cortisol levels in Carpione were consistent with cortisol plasma measured in other salmonids such as farmed Atlantic salmon broodstock [45]. In the present study, plasma cortisol levels showed a marked increase 21 days after stripping, whereas no increase was observed one day after the procedure, with respect to the PRE group [46]. Since stress-induced cortisol elevations in fish generally occur within minutes to hours following exposure to a stressor [47], the delayed increase observed in the POST21 group is unlikely to represent a direct acute response to the stripping event itself. Nevertheless, it cannot be excluded that an early transient cortisol peak occurred shortly after stripping but was not detected because of the sampling schedule adopted in the present study. Accordingly, this study was not designed to characterize the acute endocrine response to stripping, but rather to assess the physiological status of fish at short- (1 day) and longer-term (21 days) intervals following the procedure. Importantly, despite the increase in cortisol levels in the POST21 group, no concomitant alterations were detected in circulating sex steroids or ovarian morphology, suggesting that reproductive activity was maintained throughout the monitoring period. This observation suggested that, under the environmental conditions tested, the stripping procedure did not induce severe reproductive impairment. However, reproduction in female fish requires substantial energetic investment, particularly during oocyte maturation and post-ovulatory recovery [12]. Following stripping, females must undergo ovarian reorganization and tissue recovery processes, which may redirect energy allocation away from reproductive functions and toward physiological restoration [44,46]. Therefore, the delayed cortisol increase observed at 21 days may reflect the metabolic and physiological costs associated with reproductive recovery rather than the direct effects of stripping alone. In this context, the contribution of other temporal and reproductive factors occurring during this period cannot be excluded, including reproductive progression, post-ovulatory physiology and seasonal effects. This hypothesis is supported by findings in other salmonids; for instance, a similar post-ovulatory increase in glucocorticoids has been documented in Atlantic salmon, corroborating the link between metabolic restoration and corticosteroid regulation [48].

The hormonal stress profile observed in the present study was further supported by a parallel increase in antioxidant enzyme activities, suggesting the activation of a delayed systemic physiological response in the weeks following stripping. Antioxidant defense systems generally require transcriptional activation and enzyme synthesis and therefore are considered indicators of longer-term physiological adjustments rather than immediate stress responses [49]. In fish, oxidative stress may arise from multiple endogenous and exogenous factors, including temperature fluctuations, hypoxia, nutritional status, handling, reproductive effort, and alterations in energy homeostasis [50,51]. Increased metabolic activity and endocrine changes associated with reproduction and post-spawning recovery may enhance the production of reactive oxygen species [52], potentially leading to oxidative damage, including protein denaturation, lipid peroxidation, cellular dysfunction, and apoptosis when antioxidant defenses are overwhelmed [50,51].

Although antioxidant enzymes are primarily localized in metabolically active tissues such as liver, muscle, and gills, their measurement in plasma represents a useful indicator of systemic oxidative status and whole-organism physiological disturbance [53,54]. In this context, the increased antioxidant activities observed in the POST21 group, and to a lesser extent in POST1 for GPx alone, likely reflect adaptive physiological responses aimed at counteracting elevated oxidative pressure [55,56]. The simultaneous elevation of cortisol and antioxidant defenses suggests the activation of a delayed physiological response associated with reproductive recovery, rather than an early post-procedural stress reaction to the stripping method. These findings are particularly relevant considering the prolonged reproductive season of *Salmo carpio*, which requires repeated broodstock manipulation under captive conditions. In fact, the delayed cortisol and oxidative responses observed in the present study suggest that the physiological consequences of reproduction and handling may persist longer than typically considered in hatchery management protocols for other salmonid species [57,58,59]. Consequently, farming practices for Carpione should account for potential delayed stress responses by minimizing unnecessary handling during the post-spawning recovery period. Strategies providing adequate recovery intervals and limiting repeated manipulations may help mitigate physiological stress and preserve both animal welfare and reproduction throughout the breeding season.

## 5. Conclusions

The present study provided novel insights into the reproductive physiology and welfare status of captive *Salmo carpio* females possibly related to stripping procedures. Although no significant alterations were observed in reproductive hormones, ovarian histology indicated the persistence of ovarian activity throughout the examined period, with no evident histopathological lesions in the sections examined. The delayed increase in cortisol levels and antioxidant enzyme activities suggested the occurrence of longer-term physiological adjustments associated with post-spawning recovery. These findings suggested that ovarian reproductive activity was maintained during the post-stripping period, while highlighting the occurrence of physiological changes at 21 days that warrant consideration in broodstock management, and that captive reproductive management may still impose a physiological burden that should not be overlooked in conservation aquaculture programs.

Given the prolonged reproductive season of *S. carpio*, future studies should further investigate the temporal dynamics of stress responses by including earlier and more frequent post-stripping sampling points, as well as additional welfare-related biomarkers and behavioral assessments. Moreover, the development of species-specific management protocols aimed at reducing handling frequency, improving environmental conditions, optimizing recovery periods, and minimizing crowding may help mitigate stress and oxidative imbalance, thereby improving both animal welfare and reproductive efficiency in captive breeding programs for this endangered species.

## Figures and Tables

**Figure 1:**
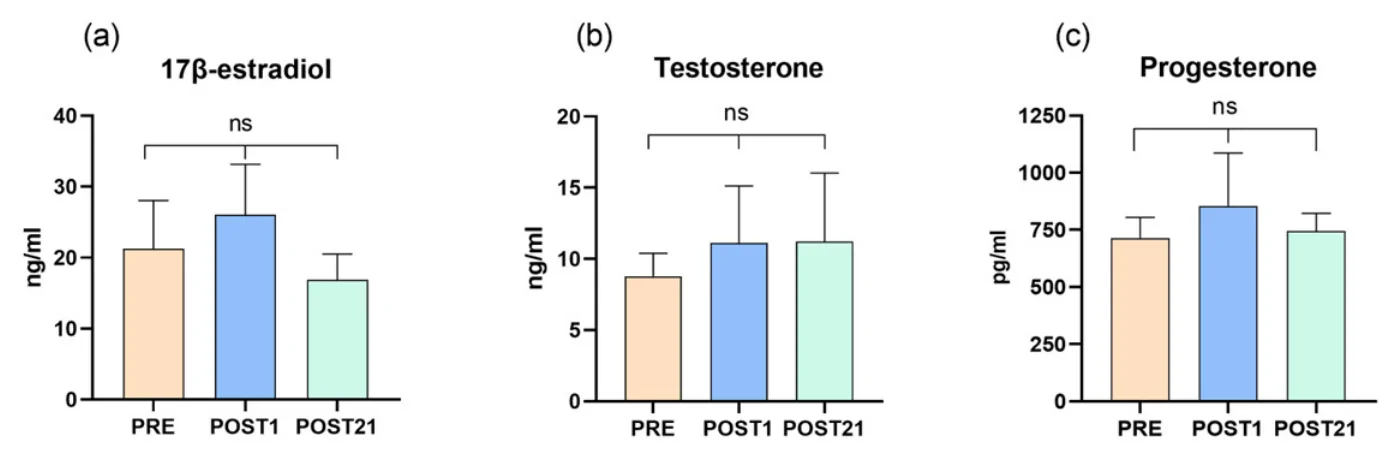
Plasma concentrations of steroid hormones measured in the plasma of female Carpione at three reproductive time points: PRE (pre-stripping), POST1 (1 day after stripping) and POST21 (21 days after stripping). (**a**) 17β-estradiol, (**b**) testosterone, and (**c**) progesterone. Values are reported as mean ± SD (*n* = 3 tanks—mean of 5 fish per tank). Abbreviations: *ns*, no significant differences among experimental groups.

**Figure 2:**
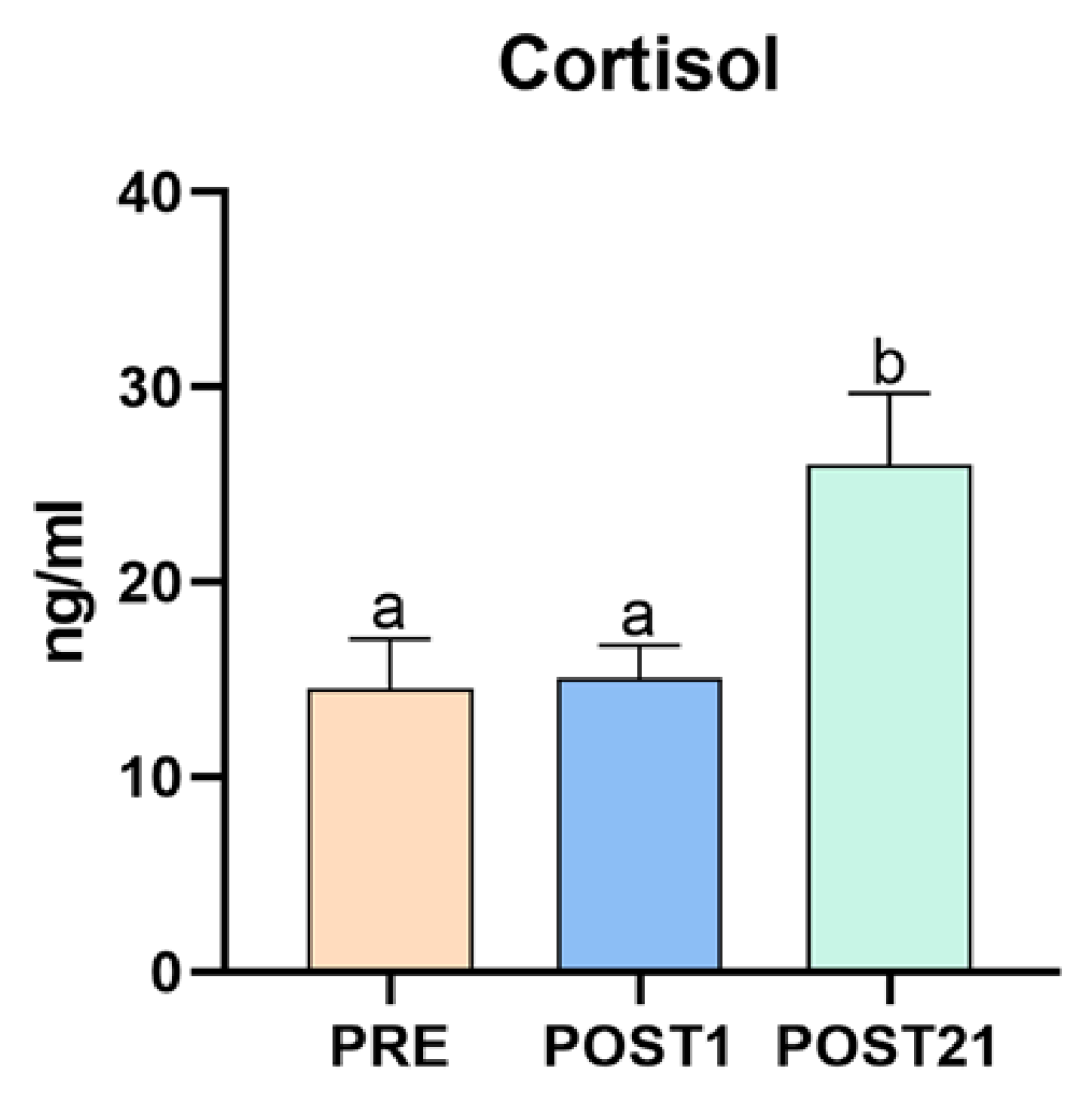
Cortisol levels (ng/mL) measured in plasma of female Carpione at three reproductive time points: PRE (pre-stripping), POST1 (1 day after stripping) and POST21 (21 days after stripping). Values are reported as mean ± SD (*n* = 3 tanks—mean of 5 fish per tank). Different letters (a–b) indicate statistically significant differences among experimental groups (*p* < 0.05).

**Figure 3:**
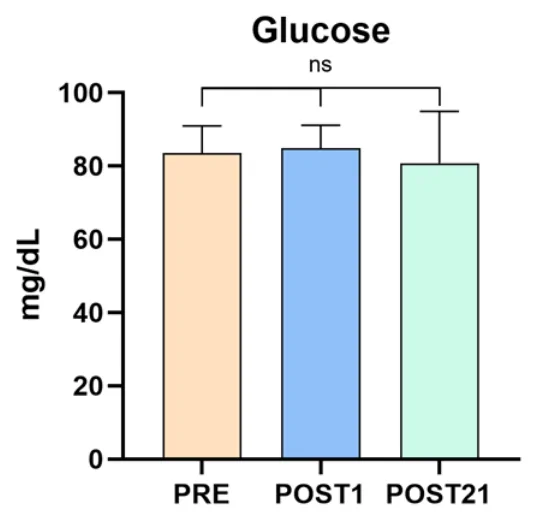
Glucose concentrations (mg/dL) measured in plasma of female Carpione at three reproductive time points: PRE (pre-stripping), POST1 (1 day after stripping) and POST21 (21 days after stripping). Values are reported as mean ± SD (*n* = 3 tanks—mean of 5 fish per tank). Abbreviations: *ns*, no significant differences among experimental groups.

**Figure 4:**
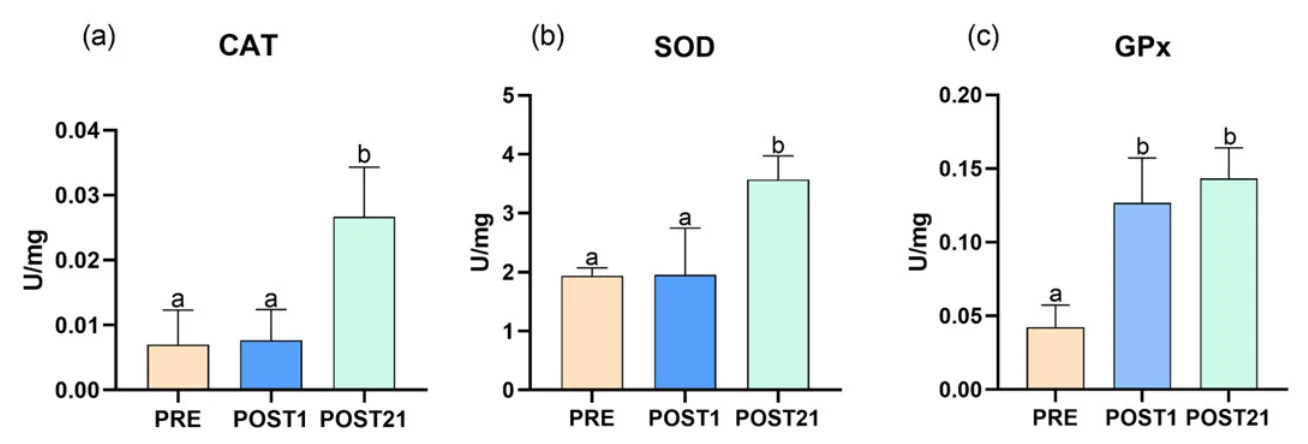
(**a**) catalase (CAT), (**b**) superoxide dismutase (SOD), and (**c**) glutathione peroxidase (GPx) activity (U/mg protein) measured in plasma of female Carpione at three reproductive time points: PRE (pre-stripping), POST1 (24 h after stripping) and POST21 (21 days after stripping). Values are reported as mean ± SD (*n* = 3 tanks—mean of 5 fish per tank). Different letters (a–b) indicate statistically significant differences among experimental groups (*p* < 0.05).

**Figure 5:**
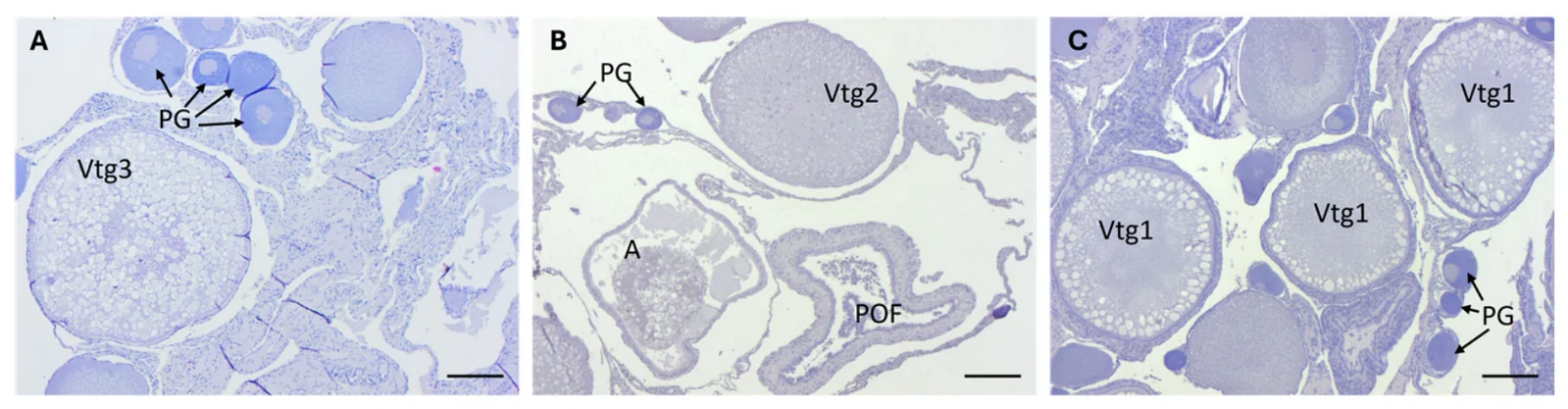
Representative images of female Carpione ovaries from the experimental groups: (**A**) PRE, (**B**) POST1, and (**C**) POST21. Abbreviations: Vtg1, primary vitellogenic oocyte; Vtg2, secondary vitellogenic oocyte; Vtg3, tertiary vitellogenic oocyte; PG, primary growth oocyte; A, atretic oocyte; POF, post-ovulatory follicle. Scale bars: 200 µm.

**Table 1:** General severity grading scale according to the OECD Guidance Document No. 123 (2010), Section 3.3.

Severity Grade	Description	Discrete Changes	Multifocal or Diffusely Distributed Alterations
Not Remarkable	This grade is used if there are no findings associated with a particular diagnostic criterion.	—	—
Grade 1: Minimal	Ranging from inconspicuous to barely noticeable, but so minor, small, or infrequent as to warrant no more than the least assignable grade.	For discrete changes, grade 1 is used when there are fewer than 2 occurrences per section.	For multifocal or diffusely distributed alterations, this grade is used for processes where ≤20% of the tissue in the section is involved.
Grade 2: Mild	A noticeable feature of the tissue.	For discrete changes, grade 2 is used when there are 3–5 occurrences per tissue section.	For multifocal or diffusely distributed alterations, this grade is used for processes where 20–50% of the tissue in the section is involved.
Grade 3: Moderate	A dominant feature of the tissue.	For discrete changes, grade 3 is used when there are 6–8 occurrences per tissue section.	For multifocal or diffusely distributed alterations, this grade is used for processes where 50–80% of the tissue in the section is involved.
Grade 4: Severe	An overwhelming feature of the tissue.	For discrete changes, grade 4 is used when there are more than 9 occurrences per tissue section.	For multifocal or diffusely distributed alterations, this grade is used for processes where >80% of the tissue in the section is involved.

## Data Availability

The raw data are not publicly available while the project is ongoing. Following completion of the project (2028), they may be made available by the corresponding author upon reasonable request, subject to the applicable agreements with the project partners.

## Abbreviations

The following abbreviations are used in this manuscript:

HPG	Hypothalamus-pituitary-gonadal
HPI	Hypothalamus-pituitary-interrenal
ELISA	Enzyme-linked Immunosorbent Assay
SOD	Superoxide dismutase
U	Unit
CAT	Catalase
GPx	Glutathione peroxidase
H&E	Hematoxylin and eosin Y
SD	Standard deviation
